# Development of a Nuclear Fuel Dissolution Monitor Based on Raman Spectroscopy

**DOI:** 10.3390/s24020607

**Published:** 2024-01-18

**Authors:** Robert Lascola, Patrick E. O’Rourke, David M. Immel

**Affiliations:** Savannah River National Laboratory, Aiken, SC 29803, USA

**Keywords:** Raman, process monitoring, nuclear material processing

## Abstract

The processing of spent nuclear fuel and other nuclear materials is a critical component of nuclear material management with implications for global security. The first step of fuel processing is dissolution, with several charges of fuel sequentially added to a batch of solvent. The incomplete dissolution of a charge precludes the addition of the next charge. As the dissolution takes place in a heated, highly corrosive and radiological vessel, direct monitoring of the process is not possible. We discuss the use of Raman spectroscopy to indirectly monitor dissolution through an analysis of the gaseous emissions from the dissolver. Challenges associated with the implementation of Raman spectroscopy include the composition and physical characteristics of the offgas stream and the impact of operating conditions. Nonetheless, we observed that NO_2_ concentrations serve as a reliable indicator of process activity and correlate to the amount of fuel material that remains undissolved. These results demonstrate the promise of the method for monitoring nuclear material dissolution.

## 1. Introduction

### 1.1. Background

The Savannah River Site (SRS) H-Canyon processing facility is used to dissolve spent nuclear fuels (SNF) and other nuclear materials as the first step in nuclear waste disposition pathways [1,2,3]. While the fuels that are dissolved have a variety of properties (cladding material, aspect ratios, and shapes), the general dissolution process is the same for all of them. Fuels are lowered into a perforated steel insert inside a large vessel containing concentrated nitric acid and a mercury catalyst. The insert has multiple wells of a number and dimension that depend on the type of fuel being dissolved. Depending on shapes of the fuel and the insert, the fuel may be either partially or completely submerged in the liquid. The vessel is closed and brought to near-boiling temperatures, promoting the dissolution of the cladding and eventually of the nuclear material contained within. Once the dissolution is complete, the vessel is allowed to cool, the lid is opened, and the level of fuel fragments is measured via mechanical probing using a remotely operated crane. Direct observation of the fragment level, such as with a submersible camera, is not practical given the nature of the solution (concentrated nitric acid, highly opaque and radioactive). Fuel fragment thresholds are defined to ensure that new fuel elements will fit into the dissolver and that nuclear safety criteria are satisfied [1]. If the fuel is found to be sufficiently dissolved, the next set of fuel is introduced. Depending on the type of fuel and the capacity of the dissolver, this process is performed up to five times before the solution is removed from the vessel and sent elsewhere in the facility for further processing.

The duration of the dissolution of each charge is defined by process history. It can range from 28 to 60 h, increasing with each charge introduced to the batch. This approach to processing is susceptible to inefficiencies that arise from incomplete dissolution, which can occur either because some components of the material dissolved more slowly than expected [4], or because parts of the material did not descend into the nitric acid. If remaining material is found to be in excess of a predetermined limit, extended dissolution is performed by resealing and reheating the dissolver for 12–24 h. The dissolver is then cooled and re-probed to confirm that fragments are at an acceptable level. An accumulation of extended dissolution scenarios creates delays in processing large amounts of material. In other cases, dissolution may be complete before the prescribed time, and if that scenario is detected, processing efficiency could increase through simply stopping earlier.

### 1.2. Dissolution Chemistry

A brief summary of the dissolution chemistry for aluminum-clad fuels [1] is as follows. On a molar basis, the bulk of the chemistry is associated with Al dissolution in concentrated (~4 M) nitric acid [5]:

Al + 3.75 HNO_3_ (+ Hg^2+^ catalyst) →                        Al(NO_3_)_3_ + 0.225 NO + 0.15 N_2_O + 0.11 N_2_ + 1.9 H_2_O + 190 kcal/mol Al (1)

The amount of nitric acid consumed and the composition of the evolved gas vary with acidity in a manner consistent with the reducing strength of the solution [6]. For example, the Al:HNO_3_ stoichiometric ratio varies from 1:4 at 2M HNO_3_ to 1:3.25 at 8 M HNO_3_. Also, an increase in the amount of Hg^2+^ catalyst will favor increased reduction, favoring N_2_O production. Importantly for this analysis, these variations do not lead to a change in the identities of the products.

The stoichiometry of the dissolution of uranium in nitric acid is not thoroughly agreed upon [7] and may be dependent on the nitric acid concentration in the solution. Based on [7], the most likely reactions are either of the two listed below:

3 UO_2_ + 8 HNO_3_ → 3 UO_2_(NO_3_)_2_ + 2 NO + 4 H_2_O (2a)

U + 4 HNO_3_ → UO_2_(NO_3_)_2_ + 2 NO + 2 H_2_O (2b)

NO_2_ may be produced from NO at nitric acid concentrations < 6.7 M [7]:

2 NO (dissolved) + HNO_3_ + H_2_O → 3 HNO_2_ (3a)

HNO_2_ + HNO_3_ → 2 NO_2_ + H_2_O (3b)

The gas passes through a condenser, which recovers nitric acid through the conversion of NO_x_ gases [1,8]:

2 NO + O_2_ → 2 NO_2_ (4a)

3 NO_2_ + H_2_O → 2 HNO_3_ + NO (4b)

The temperature of the gas leaving the dissolver is limited by the operating temperature of the dissolver, which is <105 °C [1]. After interaction with the cooling coils in the condenser, the temperature is less than 60 °C. This temperature is insufficient to promote the decomposition of N_2_O [9]. There are no known materials in the condenser that could act as catalysts. Therefore, N_2_O is expected to pass through the converter.

The offgas is next directed through a heated reactor containing silver nitrate-coated berl saddles, which trap radioactive iodine. A probable reaction is thought to be as follows [1]:

6 AgNO_3_ + 3 I_2_ + 3 H_2_O → 5 AgI + AgIO_3_ + 6 HNO_3_ (5)

with the reactor maintained between 175 and 190 °C. Finally, the offgas passes through a particulate filter (steam-heated glass wool) before being emitted from the stack. The sampling point for the offgas occurs after the particulate filter.

Another factor in the consideration of the chemical composition of the offgas is the solubility of the dissolution products in the nitric acid solutions. Both N_2_O and NO have limited solubility in nitric acid, but NO_2_ (or more specifically the dimer N_2_O_4_, which is present in an equilibrium with NO_2_) readily absorbs in nitric acid solutions [10,11]. This results in a baseline NO_2_ presence in the offgas stream when the solution is sparged and purged with air.

### 1.3. Use of Raman Spectroscopy for Dissolution Monitoring

These reactions indicate a number of species that could be proxy indicators of active dissolution. N_2_, N_2_O, NO, NO_2_, H_2_, and H_2_O are produced, and O_2_ is consumed. Raman spectroscopy was chosen as a monitoring method due to its sensitivity to these species. It has been used for a similar purpose for laboratory flowsheet dissolution studies [2,3,4]. With respect to use in the processing facility, Raman spectroscopy’s compatibility with optical fibers permits a more advantageous location for the instrument and reduces potential worker exposure to hazardous conditions. These advantages have been recognized by many laboratories that have demonstrated the suitability of Raman-based monitoring of nuclear materials, especially in solution [12,13]. Raman-based gas monitoring has been demonstrated in applications such as the measurement of I_2_ gas emitted from molten salt reactors [14] and trace gas detection in natural gas streams [15]. Raman spectroscopy has also been used for the long-term monitoring of hydrogen isotope distributions in the Karlsruhe Tritium Neutrino project [16]. The potential of Raman spectroscopy to monitor the hydrochlorination reaction of the Zircex process has recently been discussed [17]. However, to our knowledge, this is the first report of the use of Raman spectroscopy in a nuclear fuel material processing facility to monitor the dissolution of nuclear materials via the measurement of the associated offgas constituents.

## 2. Materials and Methods

**Figure 1:**
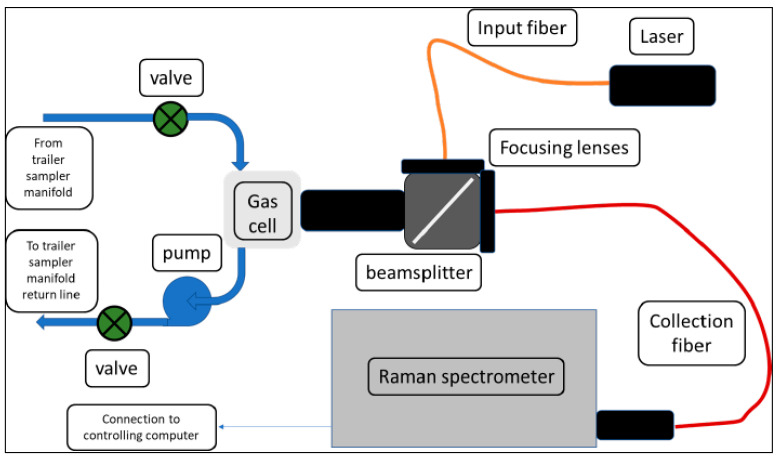
Schematic diagram of Raman instrumentation.

**Figure 2:**
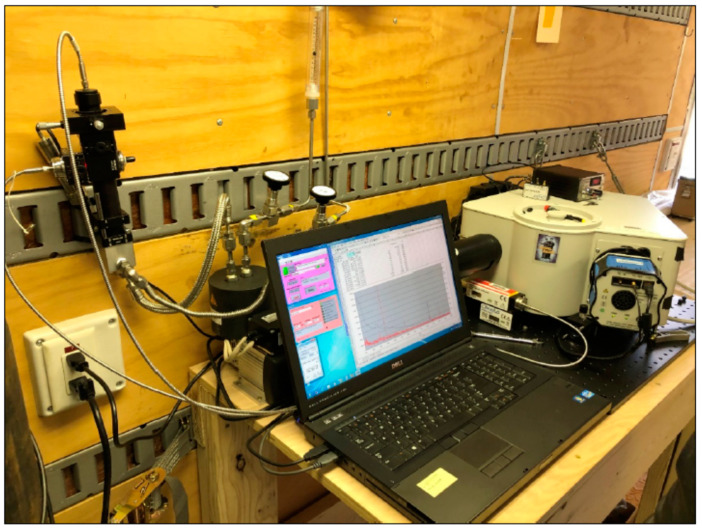
Raman instrumentation installed in the field monitoring location.

The general scheme is shown in Figure 1, and a picture of the installed unit is shown in Figure 2. Additional component details can be found in Appendix A. Light from a solid-state diode laser is coupled into an optical fiber. Either a 532 nm (300 mW) or a 640 nm (500 mW) laser is used. The emerging light is collimated, passed through a 45° angle of incidence bandpass filter, and focused into a gas cell. The Raman signal from the sample is collected in the backscattering geometry, reflected by the bandpass filter (removing the bulk of the Rayleigh-scattered excitation light), and focused into a 6-around-1 fiber bundle. The spectrometer and detector are controlled with a hybrid program that utilizes manufacturer drivers and communication protocols controlled by Visual Basic macro programs operating within an Excel spreadsheet written at SRNL.

The focusing optics and gas cell are shown in Figure 2 (upper left of the picture). All components are arranged using an optical cage mount system. The gas cell was constructed by SRNL from a machined steel block, with a volume of approximately 30 mL. Gas inlet/outlet lines are braided stainless steel, with pipe thread connections to the block and adapters allowing the use of compression fittings for the lines. The gas cell does not incorporate any retroreflection optics, waveguides, or other means to extend the sampling volume or to improve the collection of the Raman signal [15,18,19]. These improvements were not necessary for the present application but could be added if required.

The equipment and cell are located in an analytical trailer located outside H-Canyon, with an external manifold that allows for the connection of the single gas input line to exhaust lines associated with different dissolvers. The sampled gas stream is hot, acidic, condensing, and potentially radiologically contaminated. The external manifold includes a condensation pot to reduce the moisture content, which is needed to reduce the risk of inaccurate measurements associated with moisture condensation on the cell windows. The dried stream passes through a high-efficiency particulate air (HEPA) filter to remove particulates and reduce potential radiological contaminants. (The HEPA filter is not expected to adsorb gaseous components). The trailer has a pump to ensure the offgas is adequately pulled through the manifold and into the trailer. Once in the trailer, the primary gas stream is split into several secondary lines. The line for the Raman instrumentation includes a secondary pump to maintain flow. Wetted materials in the pump and fittings are temperature- and corrosion-resistant. The pump is vibrationally isolated from the rest of the equipment through the use of flexible stainless steel tubing.

For each spectrometer configuration (see Table A1), the instrument is initially calibrated for wavelength using a Ne pen lamp, then for Raman shift using cyclohexane. The cyclohexane cuvette holder is also rail-mounted and can be exchanged with the gas cell without misaligning the rest of the optical path. Continued validation of the calibration is made against the 2331 cm^−1^ N_2_ line, which is continually present in these gas streams, with linear offset corrections as needed. The efficacy of this approach is discussed below.

The process for calibrating spectral data to yield gas concentrations has been described previously [20]. Molecular response functions (MRFs) are determined for each analyte by assuming Gaussian peak shapes and literature values for Raman shifts, relative intensities, and widths for each transition of the molecule. A spectrometer response function (SRF) is determined to account for variations in grating efficiency, detector response, and other factors. The product of the MRF and SRF generates a pseudo-spectral weighting function for each analyte that is subsequently crossed with a sample spectrum to generate an intensity score for each analyte. Note that the weighting function and spectra are smoothed and processed to the second derivative to remove background contributions from fluorescence, if present, and stray light. The vector of the analyte scores is then multiplied by a matrix that accounts for the relative Raman cross-sections of each analyte, with off-diagonal elements providing corrections for overlapping peaks. Integration time and laser power provide linearly proportional changes to signal intensity but affect all peaks equally. The absolute Raman signal is also proportional to the sample line pressure, which was observed to vary between 75 and 95 kPa. Due to these effects, concentrations are reported as percent values relative to those of the components that were detected in the stream. 

## 3. Results

### 3.1. Instrument Stability

**Figure 3:**
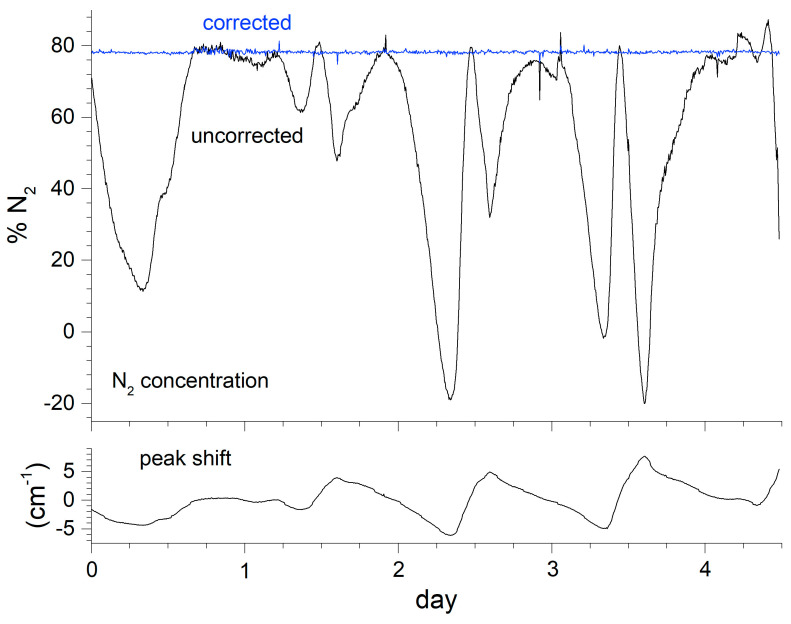
Effect of uncorrected instrument drift on N_2_ measurements.

Achieving consistent unattended measurements in a process facility usually requires accounting for fluctuating environmental conditions. Here, the instrument was located in a small trailer parked adjacent to the facility. Although air-conditioned, the trailer temperature demonstrated diurnal fluctuations, leading to Raman shift offsets. The N_2_ peak at 2331 cm^−1^ was chosen as a reference peak since that species was present for all samples measured in this application. The necessity of the correction is demonstrated in Figure 3 for a 4.5 day period where air was sampled. The peak position changes by nearly 14 cm^−1^. Uncorrected, the predicted N_2_ concentration swings wildly as the spectral overlap with the model peak function fluctuates. The blue curve shows the consistency of the concentration reading after the correction. 

**Figure 4:**
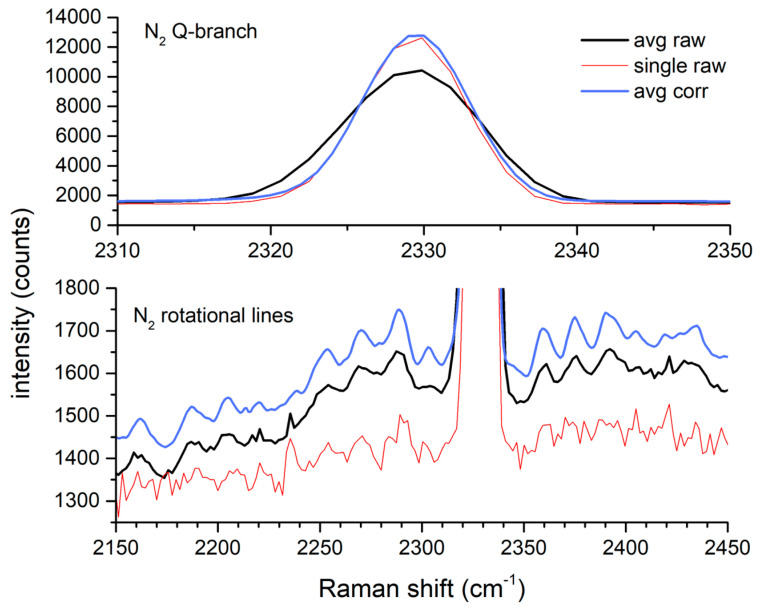
Effect of shift correction on averaged N_2_ spectra.

The effectiveness of the drift correction is also observed through the quality of the N_2_ peak over this period. Figure 4 shows that the average of the corrected spectra has a very similar peak height and width to those of a single raw spectrum that coincidentally had a minimal shift. In contrast, the average of the uncorrected spectra over the same period is broader and of a lesser intensity. The lower half of the figure shows, at an expanded scale, the N_2_ ro-vibrational Raman lines from the same figure. While these lines are apparent in the average of the uncorrected spectra, they have greater contrast and better signal to noise after drift correction.

### 3.2. Instrument Performance

**Figure 5:**
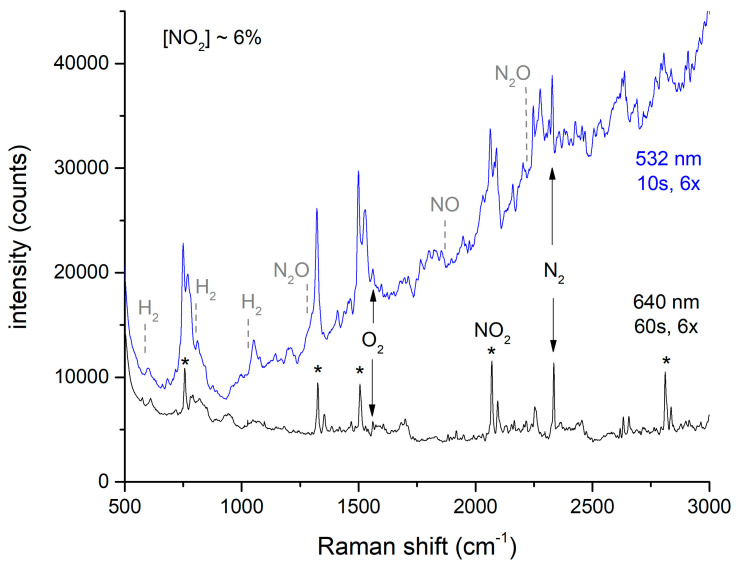
Dependence of Raman spectra of offgas during peak evolution. See text for explanation of asterisks.

As noted above, offgas spectra were taken with either 532 or 640 nm excitation. For 532 nm excitation, there is a nominal 2× signal improvement expected due to the 1/λ^4^ Raman scattering intensity dependence [21]. For NO_2_, there are absorbance lines at 532 nm [22] that lead to resonance enhancement as well as a large background of fluorescence and highly overlapping lines that complicate spectral analysis for other species, as suggested in Figure 5.

This spectrum suggests that the only species present in the offgas are N_2_, O_2_, and NO_2_. The multiplicity of peaks observed with both excitation wavelengths are due solely to NO_2_. (The strongest NO_2_ peaks that are used for quantitation are noted with asterisks). Expected positions for peaks arising from H_2_, NO, and N_2_O are noted above with dashed lines, but these peaks do not appear in the spectra. This was even true for integrated spectra recorded during the entirety of the peak emission period where NO_2_ emission was estimated at ≥1%. This spectrum in shown in Figure S1 in the Supplementary Materials. In contrast, all of these species were observed during laboratory dissolution studies, where the gases were measured immediately after emerging from the reaction vessel [2,3,4]. As the gas sampling system is not expected to alter the composition of the stream, the possibility of additional chemistry occurring in the H-Canyon offgas system should be considered. Section 4.2 further discusses the observed offgas chemistry.

The limits of detection (LOD) depend on both the excitation wavelength and the amount of NO_2_ present. When present, the NO_2_ produces a background signal that potentially interferes with the peaks of the other species. Table 1 shows the LODs for the combinations of these conditions. The LOD estimates are based on 3.3 times the standard deviation of instrument readings for an extended period of time [23]. The estimates in air are based on spectra obtained when there was no nitric acid in the dissolver tank and only air was expected in the offgas line. The estimates at peak offgas emission assume that N_2_O, NO, and H_2_ are not detectable at periods during the peak emission of NO_2_, as suggested in Figure 5 and Figure S1. Note that the shorter wavelength advantage of the 532 nm laser is somewhat offset by the higher power of the 640 nm laser and the longer integration times (10 s for 532 nm and 60 s for 640 nm, with 6× averaging for each).

Qualitatively, the limits of detection for these species in air are similar for each excitation wavelength. The power and averaging chosen for 640 nm excitation outweigh the wavelength advantage for H_2_, NO, and N_2_O, while the resonance enhancement for NO_2_ at 532 nm is an additional advantage for that species. The LODs degrade during peak offgas emissions due to the increased NO_2_ background, with the degradation being more severe for 532 nm excitation. These results point towards the use of 640 nm excitation in this application. 

### 3.3. Process Monitoring Results

During the evaluation of the Raman offgas monitor, 15 fuel dissolutions were observed, using either 532 or 640 nm as the excitation wavelength. These covered two Al-clad types, material test reactor (MTR) and high-flux isotope reactor (HFIR) fuels. The MTR and HFIR have different shapes (long/skinny rods versus squat, concentric nested cylinders). The fuels were processed in different dissolvers within H-Canyon. On some runs it was observed that the dissolver had not been sealed properly. A summary of the runs and comments on processing and monitoring are provided in Table S1 of the Supplementary Materials.

**Figure 6:**
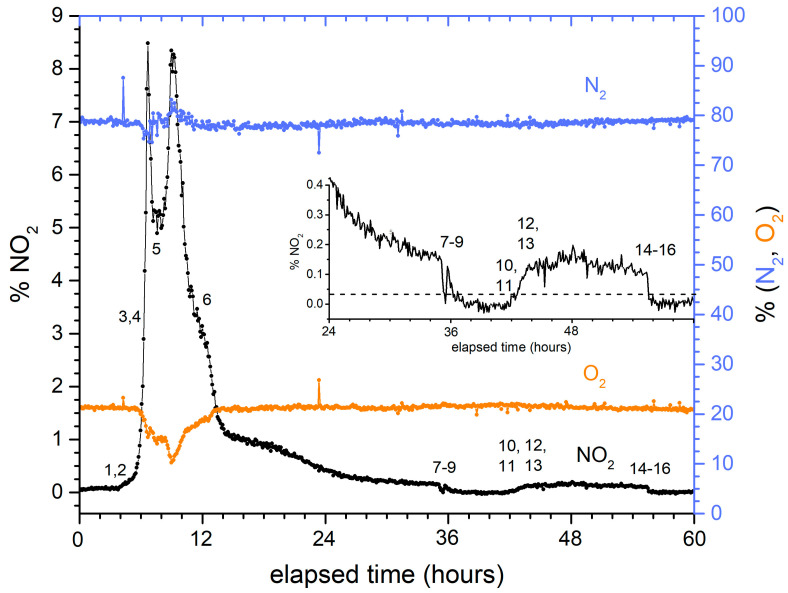
Typical time evolution profiles for NO_2_ (left axis), N_2_, and O_2_ (right axis) for nuclear fuel dissolution.

Despite these differences, the signatures of NO_2_, N_2_, and O_2_ were qualitatively similar for both fuels and for any position withing the charging sequence. Figure 6 shows a representative evolution, in this case for the dissolution of a HFIR fuel element. Notably, changes in the NO_2_ concentration can be correlated with specific processing actions, as described in Table 2. The initial rise in %NO_2_ (points 1–4) occurs when the temperature of the dissolver solution increases as steam is introduced to the heating coils in the dissolver. When a mercury catalyst was added (5–6), a second, more prolonged NO_2_ evolution was measured. NO_2_ emissions slowly decreased until the end of the prescribed dissolution time (36 h for this run) was reached and the dissolver solution was cooled (7–9). For this run, mechanical probing showed that excess solid fragments remained in the dissolver. Thus, the solution was reheated (10–13) and a second dissolving period pursued. Finally, the dissolver was cooled again (14–16). The growth of N_2_ and consumption of O_2_ can be observed during peak activity; at other times, these concentrations were indistinguishable from air within the precision of the measurement. The inset of Figure 6 shows an expanded view of the tail end of the dissolution. For this processing run, probing indicated that an excessive amount of material remained in the dissolver at the end of the primary dissolution (time ~36 h), and that an extended dissolution was required to finish the process. Despite the %NO_2_ levels being near the detection limit (the horizontal line in the inset), there is a clear difference in %NO_2_ levels between active and no dissolution, and the %NO_2_ levels clearly decrease as dissolution continues. It was further observed that there is a qualitative correlation between probe height measurements and %NO_2_ measurements. These data show that %NO_2_ levels have the potential to be used quantitatively for endpoint detection.

## 4. Discussion

### 4.1. Endpoint Detection

The qualitative observations described above suggest that a more quantitative relationship between %NO_2_ readings and the amount of material in the dissolver, as measured by probe height, might be deduced. If this were accomplished, it could be possible to use the Raman offgas monitor to estimate the fragment height of the dissolver in real time without resorting to physical probing. As probing is a time-consuming step, with further costs associated with resealing and reheating the dissolver if dissolution is found to be incomplete, process efficiency could be substantially increased. 

The mechanics of the probing suggest that the resulting height measurements have several limitations that could make it difficult to obtain a fully quantitative relationship between probing height and offgas measurements. The cross-sectional dimensions of the probe are slightly smaller than the dimensions of each well. The probe is sequentially lowered “as far as possible” [1] into each well by a remotely controlled crane. Markings on the probe indicate the height of the fragments in the well. Other limitations include:

- The only probe height data come at the end of a run, and thus there is limited offgas measurement data available to establish a correlation.
- For HFIR fuel, the insert has a single well with two sections of different widths to accommodate the two cylinders for this fuel type. Probe heights are measured simultaneously for both wells in the insert, and only the highest fragment level is recorded.
- For MTR fuel, wells are measured individually, but the mechanics of probe insertion prevent the measurement of fragment heights of less than 4”. This limitation reduces the precision of the data.
- Probe height measurements do not indicate the surface area of the remaining fragments.

Furthermore, the precision of the %NO_2_ readings at the end of a run is low (~10% of value), since the readings are ≤5× the LOD. Also, process parameters that could influence the offgas production rate, such as sparge and purge rates, vessel temperature, and dissolver leak rate, are not available in real time.

Other variable factors such as dissolver/insert geometry and Raman excitation wavelength will also influence offgas production and its measurement. There is also a background level of %NO_2_ production associated with heating concentrated nitric acid with dissolved NO_2_ [11] that will depend on the nitric acid concentration and temperature. Our preliminary studies demonstrated that these variables precluded the establishment of even a semi-quantitative global prediction model for the data we collected.

However, the value of a semi-quantitative model can be seen for the smaller set of dissolver runs where those variables are controlled. This model is based on the assumption of a proportional relationship between the amount of fuel material remaining (h_probe_) and the amount of NO_2_ evolved as a consequence of dissolution:

[%NO_2_]_dissolution_ = α · h_probe_ (6)

The gas generation constant, α, will differ for each dissolver run, since, as explained in Section 1.2, NO_2_ generation depends on solution acidity, the amount of material already dissolved, and on the efficiency of the acid recovery process in the condenser. However, it is possible to use the gas measurements obtained earlier in each dissolution run to estimate the rate near the end of the run. We will derive an expression for this constant that incorporates this information. 

We have consistently observed that over 90% of the total NO_2_ produced in a run is generated before [%NO_2_]_measured_ reaches 0.5%. With most of the material dissolved by this point, acidity and other solution properties are likely to be consistent for the remainder of the run. Also, temperature, sparge rate, and other process operating parameters mentioned above are kept consistent during the run. Therefore, the average rate of gas production taken after this point is likely to be representative of the rate at the very end of the run. This average rate, R, can be estimated by adding the measurements obtained between 0.5% and 0.2% [%NO_2_], subtracting background NO_2_, and dividing by the elapsed time, t, of the measurements. A NO_2_ value of 0.2% was chosen as the terminus for this measurement period because observations consistently showed that this value would be reached before the processing run was completed.

R = (Σ [%NO_2_]_dissolution_ + Σ [%NO_2_]_background_)/t (7)

Background NO_2_ production will depend on the process history of the solution but cannot be determined from recorded processing parameters. Thus, [%NO_2_]_background_ will be related to a fitted parameter (*b*) in the model:

Σ [%NO_2_]_background_ = *b* · t (8)

The average probe height, <h_probe_>, is not measured during this interval. We assume a proportionality constant, 1/*a*, that correlates the average rate of gas production to the average probe height.

< [%NO_2_]_dissolution_ > = (1/*a*) · R · <h_probe_**>** (9)

As it is argued that the dissolution rate is consistent through the end of the run, a comparison of Equation (6) (instantaneous gas production) with Equation (9) (average gas production) yields

α = (1/*a*) · R (10)

Substituting the expression

A_bulk_ = Σ [%NO_2_]_dissolution_ (11)

into Equation (6) and transposing yields the final equation for relating the measured %NO_2_ value to the probe height, using the fitting parameters *a* and *b*:

h_probe_ = *a* · ([%NO_2_]_measured_ − *b*)/((A_bulk_ − *b* · t)/t) (12)

An example of the use of this model to analyze data is seen for the dissolution runs for five charges of a batch of HFIR fuel into a single nitric acid solution, with all runs measured at 640 nm excitation. For this fuel and dissolver insert, a probe height of 8” or less is procedurally required to proceed to the next charge in the sequence. The measured values, derived parameters, and analysis for these five runs are shown in Table 3. For these data, values of *a* = 283 in and *b* = 0.097 [%NO_2_] minimize the differences between predicted (h_predicted_) and observed (h_probe_) probe heights, with a root mean square error of 1.3 in. The fit value for *b* is consistent with observations, being between zero (as observed for a cooled dissolver) and the tail cut values. The uncertainties listed for h*_predicted_* pertain only to the variation associated with %NO_2_ readings, and do not incorporate uncertainties in h*_probe_* or the caveats listed above that introduce imprecision in the results. Given those concerns, the observed agreement is reasonable. 

Since the intended purpose of the monitor is to indicate that the dissolution threshold (h_probe_ = 8 in) has been reached, it is worth considering how this formula might be used. The last column in Table 3 shows the %NO_2_ reading that would correspond to an estimated h_probe_ of 5.4 in, which is two standard errors less than the threshold. These values are fairly consistent despite the differences in the rate of dissolution for each charge. Because A_bulk_ and t are determinable during the run and compensate at least partially for differences in process conditions, an expected %NO_2_ reading that corresponds to a 95% certainty of completion can be defined in real time via this process.

Of the other runs monitored, there was not another sufficiently large set characterized by the use of the same excitation laser, same dissolver/fuel type, and proper dissolver sealing to perform a similar analysis. (See Table S1 in the Supplementary Materials.) Future measurements and operations are planned that will be consistent in both processing and monitoring conditions. This larger set of data will be necessary in order to demonstrate that this approach can be generalized for each fuel type across multiple batches. This capability, once proven, would allow for the development of a process monitoring history. Then, the Raman spectrometer can be used for predictive process monitoring and control during a run. 

### 4.2. Material Balance Considerations

Ideally, the readings of the Raman offgas monitor, alone or in combination with other process operating data, could be interpreted to give a measure of the mass of fuel that has been dissolved. If the gas sampling point was located in the dissolver headspace, this calculation could hypothetically be performed via a quantitation of the nitrogen-containing products in Equations (1) and (2). Percent values of these gases could be converted into absolute values via the application of the air sparge and purge rates, assuming that the system is leak-free. Although the amount of nitric acid consumed to create both aluminum and uranyl nitrate is dependent on the solution’s acidity, this information is encoded in the instantaneous ratios of the product gases.

In practice, with the sampling point located downstream of the condenser and the silver nitrate reactor, any reactions associated with those elements must be considered. Figure 6 confirms that additional reactions occur during peak dissolution periods due to the observations of O_2_ deficit and N_2_ surplus relative to concentrations in air. Any conversion would likely require the use of these values in addition to the NO_2_ measurements. Still, there are several unknowns that prevent a quantitative conversion of measured gases into the mass of fuel dissolved.

Equations (3a) and (3b), which represent the conversion of NO into HNO_3_, strongly favor conversion into acid. However, it is not likely that all the HNO_3_ is returned to the dissolver. Analysis of the liquid captured in the condensing trap located on the analytical trailer sample inlet line showed the solution to be strongly acidic and to have a dark-brown color. Thus, there is an appreciable but unknown fraction of NO_x_ that does not reach the analyzer.

N_2_O is apparently destroyed before reaching the analyzer, but the specific reactions associated with that process are not known. Temperatures in the offgas line neither reach the values required for the spontaneous decomposition of N_2_O nor match the temperatures reported in the literature at which the catalyzed decomposition of or a reaction with NO could occur [9]. In a prior study at SRS, the analysis of numerous grab samples of stack effluent gas from the dissolution of aluminum-clad uranium slugs in F-Canyon also showed substantially more NO_2_ than N_2_O (70–120x during peak NO_2_ emission) [24]. However, this process removed most of the aluminum cladding with a 50–50% NaNO_3_-NaOH solution before nitric acid attack. Thus, much of the current source for N_2_O was not present in this historical example, and the results are not directly comparable (even assuming that the gas treatment was identical, which might not be the case). Recent studies [25] examining the role that the silver nitrate-coated berl saddles may have in the catalytic destruction of these species did not show any changes in the concentrations of N_2_O and NO. The reason for the loss of N_2_O remains unexplained.

Both considerations would influence the interpretation of the nitrogen balance in the basic dissolution equation (Equation (1)). The acidity of the dissolvent determines the molar ratio of N_2_O, NO, and N_2_ formed in the dissolver headspace as well as the molar ratio of the sum of these gases to the three moles of N required to convert one mole of Al into Al(NO_3_)_3_. Furthermore, if the conversion pathway of N_2_O is not known, it is uncertain whether it should be accounted for as NO_2_ or N_2_/O_2_ growth.

The studies on silver nitrate-coated berl saddles show that under laboratory conditions consistent with process parameters, H_2_ was catalytically recombined with O_2_ with high efficiency (>97% for residence times of >10 s) [25]. As noted earlier, %O_2_ levels are depressed during peak NO_2_ offgassing to a greater extent that would be expected due to displacement. A loss of O_2_ through recombination with H_2_ as shown in the studies of silver nitrate-coated berl saddles is one explanation for this observation. However, the loss of O_2_ is not a quantitative proxy for the loss of H_2_, since O_2_ is also lost during the recovery of nitric acid from NO in the condenser (see Equation (4a)). Thus, uncertainties associated with this process hinder using O_2_ loss as a means for working back to aluminum dissolution.

The potential accuracy of the estimate should also be considered. The accumulated uncertainties of the components in the calculation could exceed the precision required for the estimate to be meaningful. Uncertainties of N_2_ and O_2_ concentrations are 0.5% and 1.5% (1σ) of the reading, as described previously. These uncertainties would be increased by two because the values of interest, the difference from the measurement baseline, are obtained from the subtraction of two uncertain numbers. Based on the comparison of Raman intensities, the uncertainties of NO_2_ readings during the peak of the dissolution will be somewhere between those of N_2_ and O_2_—perhaps 1% (1σ) of the reading. The correction factor associated with the efficiency of the condenser will have an unknown uncertainty, perhaps on the order of several percent. The variation of the acidity of the dissolvent, and its effect on the ratio of species in the offgas could contribute a few percent as well. Although not all these contributions to the uncertainty are quantifiable, it is reasonable to assume that they combine toward an uncertainty of at least 5%.

One hypothetical example where the estimate of dissolved fuel would be of interest is as a confirmatory measurement that a large amount of material has not been dissolved (for example, something greater than the 36” fragment height threshold for MTR fuel remains in that dissolver). For a six-bundle charge of fuel, with each bundle being 132” long, a single fragment would comprise 4.5% of the total amount of material charged. This difference is exceeded by the likely uncertainty derived above.

## 5. Conclusions

In this paper, we have described the development and deployment of a monitor to track fuel dissolution in a nuclear material processing facility. The monitor is based on Raman spectroscopy of the dissolver offgas stream. Changes in %NO_2_ levels are observed to correlate with dissolver processing activities. A semiquantitative relationship between %NO_2_ levels at the end of the dissolution and the level of fuel fragments observed in the dissolver has been proposed and demonstrated for data obtained for one batch run of one fuel type for which consistent Raman data were obtained. This relationship is strongly dependent on fuel type (shape and material). Additional investigations are required to demonstrate that similar relationships can be developed for other fuel types and with data obtained from multiple batches. The precision of the relationship may be limited by uncontrolled processing factors. However, the initial results suggest that the relationships can be used as a basis to indicate that the fragment level in the dissolver is below a threshold required to declare that dissolution is complete. This use, if substantiated by future results, would help make processing more efficient and enhance safety by protecting nuclear criticality limits.

We also discuss the observed composition of the offgas. Several species expected to be generated during dissolution are not detected by the spectrometer. The fate of some species, such as H_2_ and NO, can be explained by reactions in elements of the offgas stack. However, the reason for the apparent disappearance of N_2_O is less clear.

## Figures and Tables

**Table 1:** Limits of detection (LODs) for offgas species and for gas samples at atmospheric pressure.

	In Air		At Peak Offgas Emission ^1^	
Species	LOD (532 nm)	LOD (640 nm)	LOD (532 nm)	LOD (640 nm)
H_2_	0.59%	0.52%	0.67%	0.60%
NO_2_	0.031%	0.041%	--- ^1^	---
NO	1.3%	0.73%	7.0%	2.8%
N_2_O	0.28%	0.23%	0.94%	0.41%

^1^ Defined by >1% NO_2_ in the offgas; LODs for NO_2_ in this regime do not apply.

**Table 2:** Operational events associated with dissolution (re. Figure 6).

Event	Description	Event	Description
1	Sparge turned on	*probed high, extended dissolution required*	
2	Steam introduced	10	Sparge turned on
3	Dissolver reached boiling	11	Steam introduced
4	Switched sparge to purge	12	Reached boiling
5	Mercury addition initiated	13	Switched sparge to purge
6	Mercury addition completed	14	Dissolution completed, steam stopped
7	Steam stopped	15	Switched purge to sparge
8	Switched purge to sparge	16	Air sparge control valve closed
9	Air sparge control valve closed		

**Table 3:** Derived values for parameters and predictions for probe height for HFIR batch.

Charge	[NO_2_]_measured_ at End of Run (%)	A*_bulk_* (%·h)	T*_bulk_* (h)	h*_probe_* (in)	h*_predicted_* (in) ^1^	[NO_2_] for “Completion” (%)
1	0.12 ± 0.01	23.4	8.0	4.5	3.3 ± 0.4	0.144
2	0.135 ± 0.02	32.7	12.4	7	6.4 ± 0.9	0.135
3	0.12 ± 0.01	43.0	15.4	1	3.6 ± 0.4	0.141
4	0.11 ± 0.02	47.9	17.7	3	2.1 ± 1.0	0.140
5	0.11 ± 0.01	30.1	9.6	0 ^2^	1.7 ± 0.4	0.147

^1^ As determined from fitting data to Equation (6) to estimate *a* and *b*. Uncertainties are propagated from the %NO_2_ values. ^2^ Reported as “not detected” by the facility.

## Data Availability

Requests for the data presented in this study are subject to review by the United States Department of Energy.

## Supplementary Materials

The following supporting information can be downloaded at https://www.mdpi.com/article/10.3390/s24020607/s1. Figure S1: Absence of observed H_2_ Raman scattering lines in spectra obtained at peak gas emission during a representative dissolver run.; Figure S2: Absence of observed N_2_O and NO Raman scattering lines in spectra obtained at peak gas emission during a representative dissolver run. Table S1: Summary of operating and monitoring conditions for dissolver runs.

## Appendix A

Component specifications are as follows. Spectrometer: iHR 320, f/4.1, 0.32 m (Horiba). 600 mm-1 grating. Detector: Syncerity 2048x512-NIR (Horiba), −75 °C. Laser: LPX-640 (Oxxius), 640 nm, or Duetto532 (RMPC Lasers), 532 nm. The seven linearly aligned fibers of the 6-around-1 collection fiber (ThorLabs) are oriented to match the entrance slit of the spectrometer. Optical filters: StopLine notch filter, RazorEdge dichroic filter, and RazorEdge long-pass edge filter at appropriate wavelengths (Semrock). Lenses: Edmund Optics. Optomechanics: ThorLabs.

Spectral acquisition parameters are shown in Table A1.

**Table A1:** Spectral acquisition parameters.

Laser Wavelength (nm)	Grating Density (mm^−1^)	Slit Width (μm)	Center Wavelength (nm)	Spectral Range (cm^−1^)	Acquisition Time (s) and Averages	Readout Rate (kHz)	Approx. Peak FWHM (cm^−1^)
532	600	69	608	365–4130	10, 6×	45	8
640	600	69	720	385–3022	60, 6×	45	6

## References

[1] Hyder M.L., Perkins W.C., Thompson M.C., Burney G.A., Russell E.R., Holcomb H.P., Landon L.F. (1979). Processing of Irradiated Enrich Uranium Fuels at the Savannah River Plant.

[2] Daniel W.E., Rudisill T.S., O’Rourke P.E., Karay N.S. (2019). Dissolution flowsheet for high flux isotope reactor fuel at SRS. Sep. Sci. Technol..

[3] Daniel W.E., Rudisill T.S., O’Rourke P.E. (2018). Dissolution of Material Test Reactor Fuel in an H-Canyon Dissolver.

[4] Daniel W.E., Rudisill T.S., Mickalonis J.I. (2020). Evaluation of the Dissolution Behavior of L-Bundle End Caps and HFIR Fuel Carriers.

[5] Wymer R.G., Blanco R.E. (1957). Uranium-Aluminum Alloy Dissolution. Ind. Eng. Chem..

[6] Rice R.W., Sarode D.V. (2001). Mercury-Catalyzed Dissolution of Aluminum in Nitric Acid. Ind. Eng. Chem. Res..

[7] Marc P., Magnaldo A., Vaudano A., Delahaye T., Schaer E. (2017). Dissolution of Uranium Dioxide in Nitric Acid Media: What Do We Know?. EPJ Nuclear Sci. Technol..

[8] Compilation of Air Pollutant Emissions Factors (AP-42), U.S. Environmental Protection Agency, Chapter 8: Inorganic Chemical Industry. https://www.epa.gov/air-emissions-factors-and-quantification/ap-42-compilation-air-emissions-factors.

[9] Groves M.C.E., Sasonow A. (2010). Uhde EnviNO_x_(R) Technology for NO_x_ and N_2_O Abatement: A Contribution to Reducing Emissions from Nitric Acid Plants. J. Integr. Environ. Sci..

[10] Tan S.P., Piri M. (2013). Modeling the Solubility of Nitrogen Dioxide in Water Using Perturbed-Chain Statistical Associating Fluid Theory. Ind. Eng. Chem. Res..

[11] Lefers J.B., van den Berg P.J. (1982). Absorption of NO_2_/N_2_O_4_ into Dilute and Concentrated Nitric Acid. Chem. Eng. J..

[12] Bryan S.A., Levitskaia T.G., Johnsen A.M., Orton C.R., Peterson J.M. (2011). Spectroscopic Monitoring of Spent Nuclear Fuel Reprocessing Streams: An Evaluation of Spent Fuel Solutions via Raman, Visible, and Near-infrared Spectroscopy. Radiochim. Acta.

[13] Tse P., Bryan S.A., Bessen N.P., Lines A.M., Shafer J.C. (2020). Review of On-line and Near Real-time Spectroscopic Monitoring of Processes Relevant to Nuclear Material Management. Anal. Chim. Acta.

[14] Felmy H.M., Clifford A.J., Medina A.S., Cox R.M., Wilson J.M., Lines A.M., Bryan S.A. (2021). On-Line Monitoring of Gas-Phase Molecular Iodine Using Raman and Fluorescence Spectroscopy Paired with Chemometric Analysis. Environ. Sci. Technol..

[15] Buric M.P., Falk J., Woodruff S., Chorpening B. (2017). Gas Phase Raman Scattering: Methods and Applications in the Energy Industry. Encyclopedia of Spectroscopy and Spectrometry.

[16] Aker M., Altenmüller K., Beglarian A., Behrens J., Berlev A., Besserer U., Bieringer B., Blaum K., Block F., Bornschein B. (2020). Quantitative Long-Term Monitoring of the Circulating Gases in the KATRIN Experiment Using Raman Spectroscopy. Sensors.

[17] Peruski K.M., Vestal B.K., Vick M., Cobble C., Johnson K.R., McFarlane J. (2023). On-Line Measurement of Hydrogen Gas Using Raman Spectroscopy for Process Gas Systems.

[18] Pearman W.F., Carter J.C., Angel S.M., Chan J.W.-J. (2008). Multipass Capillary Cell for Enhanced Raman Measurements of Gases. Appl. Spectrosc..

[19] Wen C., Huang X., Shen C. (2020). Multiple-pass Enhanced Raman Spectroscopy for Fast Industrial Trace Gas Detection and Process Control. J. Raman Spectrosc..

[20] O’Rourke P.E. (2020). Calibration of Raman Spectrometer for Gas Phase Measurements.

[21] Dudik J.M., Johnson C.R., Asher S.A. (1985). Wavelength dependence of the preresonance Raman cross sections of CH_3_CN, SO_4_^2−^, ClO_4_^−^, and NO_3_^−^. J. Chem. Phys..

[22] Vandaele A.C., Hermans C., Fally S., Carleer M., Colin R., Mérienne M.-F., Jenouvrier A., Coquart B. (2002). High-resolution Fourier transform measurement of the NO_2_ visible and near-infrared absorption cross sections: Temperature and pressure effects. J. Geophys. Res..

[23] Armbruster D.A., Pry T. (2008). Limit of Blank, Limit of Detection and Limit of Quantitation. Clin. Biochem. Rev..

[24] Villa E. (1997). Analysis of F-Canyon Effluents during the Dissolution Cycle with a Fourier Transform Infrared Spectrometer Multipass Cell.

[25] Gogolski J.M., Taylor-Pashow K.M.L., Rudisill T.S., Restivo M.L., Pareizs J.M., Lascola R.J., O’Rourke P.E., Daniel W.E. (2022). Catalytic Effects of Silver in Iodine Reactors for Dissolved Used Nuclear Fuel. Nuclear Technol..

