# Supplementary material for: Development of a Nuclear Fuel Dissolution Monitor Based on Raman Spectroscopy
Source: Sensors (Basel). 2024 Jan 18;24(2):607. doi: 10.3390/s24020607 (PMC10819358; doi:10.3390/s24020607)
Supplement: Supplementary file 1 [file sensors-24-00607-s001.zip › sensors-2780493-supplementary.pdf]

Supplemental Materials for: "Development of a Nuclear Fuel Dissolution Monitor Based on Raman Spectroscopy", by R. Lascola, P.E. O'Rourke, and D.M. Immel. To be published in *Sensors*.

Figures S1 and S2 demonstrate the extent to which Raman lines associated with H_2 (Figure S1) and N_2O and NO (Figure S2) were not observable during periods of peak gas evolution during a dissolver run. Expected positions for these analytes are indicated with arrows. The NO_2 levels for the spectra used to calculate the average spectrum were generally greater than 1%. The 1876 cm^{-1} NO peak is coincident with a small NO_2 line; however, the intensity ratio of this peak to nearby NO_2 peaks is consistent with ratios observed for spectra of NO_2 by itself. Thus, there is no visual evidence for the NO peak. This coincidence of lines is one important reason for the comparatively large LOD for NO compared to other species. Assuming that the LOD improves with the square root of the number of averages, the absence of evidence for N_2O, NO, and H_2 in the highly averaged spectra suggests that the concentrations of those species during peak dissolver emission are no greater than $1/\sqrt{112} = 0.094$x the LODs shown in Table 1 of the paper.

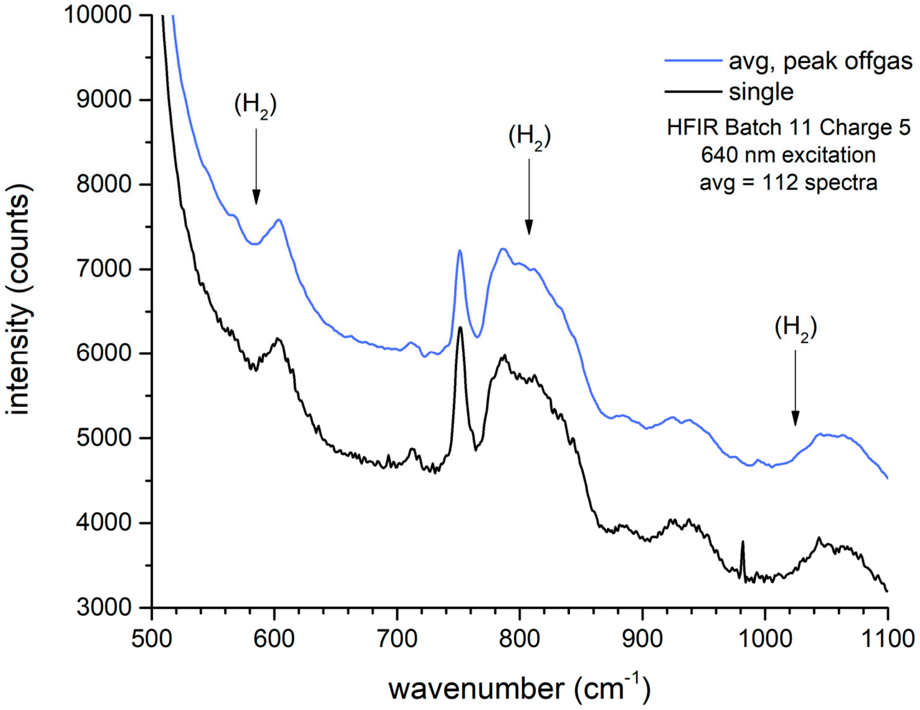

Figure S1. Absence of observed H_2 Raman scattering lines in spectra obtained at peak gas emission during a representative dissolver run.

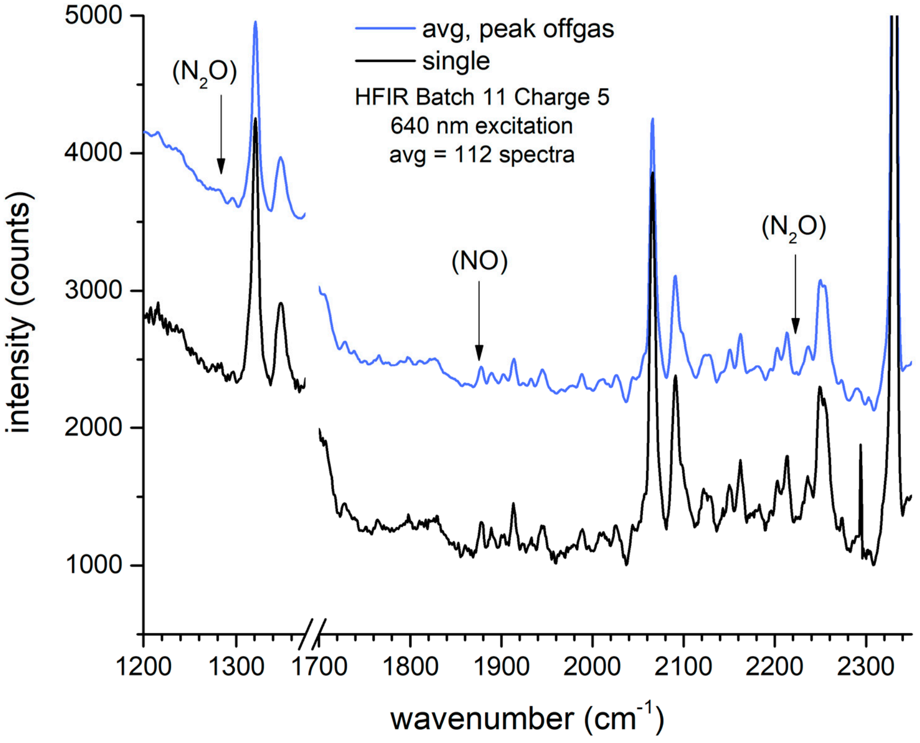

Figure S2. Absence of observed N_2O and NO Raman scattering lines in spectra obtained at peak gas emission during a representative dissolver run.

In the main text, we describe a relationship between measured %NO_2 concentrations and fuel fragment height in the dissolver. This relationship is applied to data obtained for 5 runs for which there are consistent conditions, such that the results can be reasonably compared. These conditions include Raman excitation wavelength, fuel type, dissolver identity, and dissolver condition (most specifically, the dissolver lid being leak-tight). We also state that a total of 15 dissolver runs were monitored. The other 10 runs did not have a similarly consistent set of conditions to further test the relationship between probe height and gas measurements. A summary of the operating parameters and Raman spectroscopy configuration is shown in Table S1.

Table S1. Summary of operating and monitoring conditions for dissolver runs.

Fuel Type / Batch	Charge Number	Raman Laser	Comments
MTR #22	1	640 nm	Dissolver not fully sealed; offgas diluted.
	2		Dissolver not fully sealed; offgas diluted.
	3		Dissolver not fully sealed; offgas diluted.
MTR #24	1	532 nm	Dissolver not fully sealed; offgas diluted.
	2		Dissolver not fully sealed; offgas diluted.
	3		Dissolver not fully sealed; offgas diluted.
MTR #26	1	532 nm	All systems normal.
	2		Raman instrument malfunction.
HFIR #11	1	640 nm	All systems normal.
	2		All systems normal.
	3		All systems normal.
	4		All systems normal.
	5		All systems normal.
HFIR #13	1	532 nm	All systems normal.
	2		All systems normal.
	3		Steam turned off mid-run for approx. 13h.
	4		*(not monitored)*
	5		*(not monitored)*
